# Human Neonatal Dendritic Cells Are Competent in MHC Class I Antigen Processing and Presentation

**DOI:** 10.1371/journal.pone.0000957

**Published:** 2007-09-26

**Authors:** Marielle C. Gold, Tammie L. Robinson, Matthew S. Cook, Laura K. Byrd, Heather D. Ehlinger, David M. Lewinsohn, Deborah A. Lewinsohn

**Affiliations:** 1 Department of Pulmonary and Critical Care Medicine, Oregon Health & Science University, Portland, Oregon, United States of America; 2 Portland VA Medical Center, Portland, Oregon, United States of America; 3 Department of Pediatrics, Oregon Health & Science University, Portland, Oregon, United States of America; Federal University of Sao Paulo, Brazil

## Abstract

Neonates are clearly more susceptible to severe disease following infection with a variety of pathogens than are adults. However, the causes for this are unclear and are often attributed to immunological immaturity. While several aspects of immunity differ between adults and neonates, the capacity of dendritic cells in neonates to process and present antigen to CD8^+^ T cells remains to be addressed. We used human CD8^+^ T cell clones to compare the ability of neonatal and adult monocyte-derived dendritic cells to present or process and present antigen using the MHC class I pathway. Specifically, we assessed the ability of dendritic cells to present antigenic peptide, present an HLA-E–restricted antigen, process and present an MHC class I-restricted antigen through the classical MHC class I pathway, and cross present cell-associated antigen via MHC class I. We found no defect in neonatal dendritic cells to perform any of these processing and presentation functions and conclude that the MHC class I antigen processing and presentation pathway is functional in neonatal dendritic cells and hence may not account for the diminished control of pathogens.

## Introduction

About 2 million children die each year of infectious diseases (World Health Organization Maternal Health and Safe Motherhood Programme MSM96.7, 1996). Young infants are more susceptible to severe disease from infectious pathogens than are older children and adults [1]. Specifically, infections with respiratory syncytial virus, HIV, *Streptococcus pneumoniae*, *Mycobacterium tuberculosis* (Mtb) and *Plasmodium sp.* disproportionately cause morbidity and mortality in infants [2]. The causes for this are likely to be pleomorphic, and include the reduced frequency and diminished functional capacity of neonatal T cells [3]. While studies in both humans and mice demonstrate decreased T cell function in neonates, this defect can be overcome by the administration of strong adjuvants [4]. These data suggest that the defect might rest in the priming of naïve T cells. Dendritic cells (DC) are unique in their capacity to present processed antigen to prime naïve T cells *in vivo*: CD4^+^ T cells via the MHC II pathway, and CD8^+^ T cells via the MHC I pathway, respectively [5]. The results from studies evaluating the MHC II antigen processing and presentation pathway in neonates are equivocal but current reviews suggest neonatal DC may be defective in this regard [3], [6]. However, little is known about the functionality of DC in antigen processing and presentation via the MHC I pathway in neonates. Ultimately, the most stringent functional test of DC function is their capacity to prime optimal T cell responses *in vivo*. While this is difficult to evaluate in humans, Salio et al. showed that DC from neonates could efficiently prime CD8^+^ T cells *in vitro*. However, priming was performed using DC loaded with antigenic peptide and again these DC were not tested for their ability to present processed antigen. To prime cytolytic CD8^+^ T cells, dendritic cells must first process cytosolic or exogenously-acquired antigens, load them onto newly synthesized MHC class I molecules, and transport the loaded MHC I molecules to the cell surface where they are available to stimulate CD8^+^ T cells. To date, no study has directly evaluated the capacity of human neonatal DC to process and present MHC class I antigens to CD8^+^-restricted T cells. Therefore, to address the hypothesis that the increased severity of disease associated with neonates is due to a defect in DC MHC I antigen processing and presentation, we performed a comprehensive study using monocyte-derived DC isolated from neonatal cord blood mononuclear cells (CBMC) and adult peripheral blood mononuclear cells (PBMC). We assessed the ability of DC to present antigenic peptide, present a MHC class Ib (HLA-E)-restricted antigen, process and present a MHC class Ia antigen delivered through viral infection, and finally, cross present cell-associated antigen. This thorough functional examination of neonatal DC demonstrates that these cells are fully competent APC in their ability to process and present antigen to CD8^+^ T cells.

## Results

**Figure 1:**
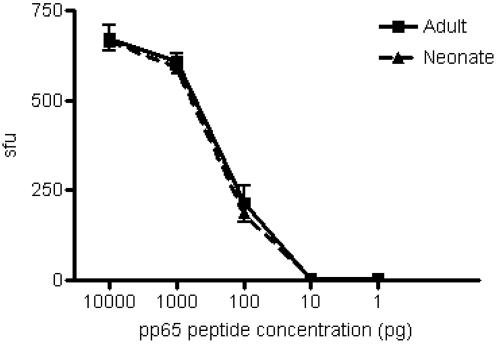
Equivalent peptide presentation of the HCMV pp65 nonamer NLVPMVATV by DC from adult and neonatal blood to HLA-A2- restricted clone CD8^+^ T cell clone D2-1-D2. The peptide NLVPMVATV was added in 10-fold dilutions (final concentration 10,000 pg/ml to 10 pg/ml) to HLA-A2^+^ monocyte-derived DC (20,000 cells/well) from adult (*n = *4) and neonatal (*n = *7) blood and incubated overnight with D2-1-D2 CD8^+^ T cell clones (10,000 cells/well). IFN-γ production was detected by ELISPOT. Linear regression analysis was used to determine that there was no statistically significant difference between adult and neonatal DC in presentation of peptide to HLA-2-restricted CD8^+^ T cells (p = 0.9882). Monocyte-derived DC (MDDC) were used as antigen presenting cells in all experiments and were derived as described in materials and methods.

To directly assess the functional capacity of DC, experimental conditions have been established in which the activation of CD8^+^ T cells is dependent on the availability of functional APC [7]. In brief, DC are titrated in the presence of excess numbers of T cell clones such that T cell activation is limited by the availability of DC. To first evaluate the MHC I antigen presentation pathway in neonatal DC, we assessed the capacity of DC to present a minimal peptide epitope to cognate CD8^+^ T cells, an assay reflective of the density of HLA-I on the cell surface. Specifically, we assessed the ability of DC to present an HLA-A2-restricted peptide to the HLA-A2-restricted, HCMV pp65_(495-503) _NLVPMVATV-specific CD8^+^ T cell clone D2 1-D2. Here, we observed equivalent presentation of the HCMV pp65 peptide over a range of concentrations by neonatal (*n = *7) and adult (*n = *4) DC (Figure 1, p = 0.9882).

**Figure 2:**
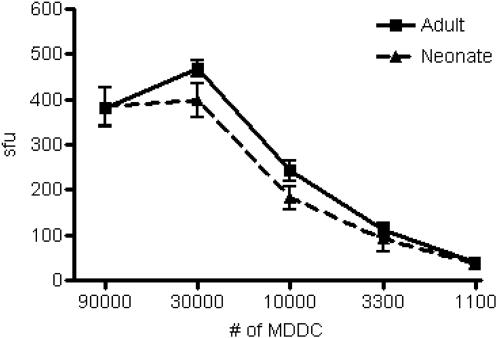
DC from adults and neonates are comparable in their ability to present the *M. tuberculosis* antigen pronase-digested cell wall to HLA-E-restricted clone D160-1-23. DC from adult (*n = *5) and neonatal (*n = *6) blood were incubated overnight with or without the pronase-treated cell wall fraction from *M.tuberculosis*. The DC were diluted over a range of concentrations (90,000 to 1100 cells/well), and incubated with the cognate CD8^+^ HLA-E-restricted clone D160-1-23 (10,000 cells/well) overnight. IFN-γ was detected by ELISPOT. There was no statistically significant difference between adult and neonatal DC in presentation of pronase-digested cell wall to HLA-E-restricted CD8^+^ T cells (p = 0.7943).

HLA class Ib-restricted responses are also associated with certain pathogens [8]. Notably, roughly fifty percent of the CD8^+^ T cell response to Mtb is comprised of non-classical MHC Ib-restricted T cells [9]. Therefore, we sought to evaluate the presentation of an MHC class Ib-restricted antigen by neonatal DC. The CD8^+^ T cell clone D160 1-23 recognizes an HLA-E-restricted antigen derived from the cell wall of Mtb [10]. Therefore, we incubated DC with pronase-treated Mtb cell wall and assessed the ability of clone D160 1-23 to produce IFN-γ. Again, neonatal DC (*n = *6) were equally capable as adult DC (*n = *5) to stimulate HLA-E-restricted CD8^+^ T cells (Figure 2, p = 0.7943).

**Figure 3:**
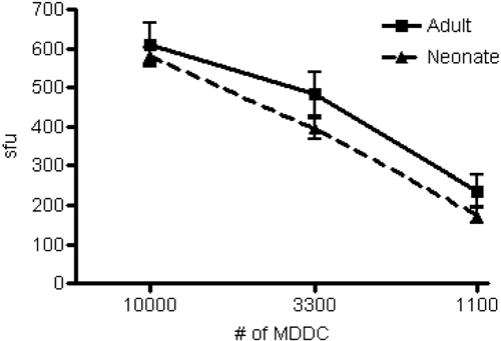
DC from adult and neonatal blood are similar in their ability to process and directly present the HLA-A2 restricted antigen HCMV pp65 epitope NVLPMVATV following recombinant vaccinia infection. HLA-A2^+^ DC from adults (*n = *11) and neonates (*n = *7) were infected with vvpp65 (moi = 1.5) overnight. Infected and uninfected DC were titrated over a range (10,000 to 1,100 cells/well) and incubated overnight with pp65-specific CD8^+^ T cell clone D2-1-D2 (10,000 cells/well). IFN-γ production was detected by ELISPOT. HLA-A2^−^ DC from both adults and neonates did not induce any IFN-γ production by CD8^+^ T cell clone D2-1-D2. There was no statistically significant difference between adult and neonatal DC in processing and directly presenting antigen following recombinant vaccinia infection (p = 0.6489).

Given the enhanced susceptibility of neonates to viral infection, we postulated that neonatal DC may be deficient in their ability to process intracellular antigen in the context of HLA-I. To address this possibility, we infected DC with vaccinia virus expressing the HCMV antigen pp65. We then titrated the DC over a range and assessed the ability of cognate T cell clone D2 1-D2 to produce IFN-γ by ELISPOT. Figure 3 shows no significant difference in the ability of neonatal DC (*n = *7) compared to adult DC (*n = *11) to present the vaccinia-delivered pp65 antigen to HLA-A2-restricted CD8^+^ T cell clone D2-1-D2 (p = 0.6489).

**Figure 4:**
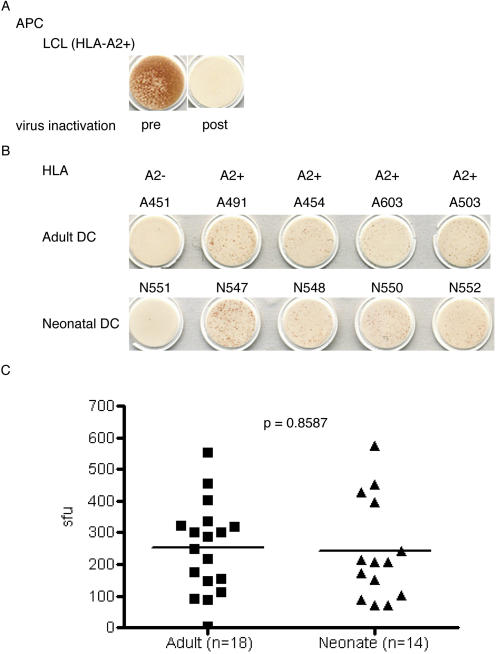
DC from adults and neonates are equivalent in their ability to cross present cell-associated antigen. **4A**. To confirm inactivation of vaccinia virus, HLA-A2^+ ^LCL (30,000 cells/well) were co-incubated for 24 hrs with vvpp65-infected HLA-A2^−^ LCL (60,000 cells/well) prior to (left well) or after (right well) heat-inactivation (30 minutes at 56C) and UV-treatment (200mJ^2^). Direct presentation was then detected by IFN-γ ELISPOT after an overnight incubation with CD8^+^ T cell clones D2 1-D2 (10,000/well), specific for the HLA-A2-restricted antigen HCMV pp65. **4B.** Representative ELISPOT wells of the cross presentation assay are shown. DC (30,000/well) from five individual adult (top row) or neonatal donors (bottom row) were incubated overnight with cell-associated antigen, namely, vvpp65-infected LCL (60,000/well) that were heat (30 minutes at 56C) and UV-treated (200 mJ^2^). CD8^+^ T cell clones D2 1-D2, specific for the pp65 antigen, were added (10,000 cells/well) and IFN-γ was detected the following day by ELISPOT. Only HLA-A2^+^ DC cross presented the pp65 antigen (right 4 columns) and HLA-A2^−^ DC (left column) never cross presented the antigen. HLA-A2^+^ DC incubated with uninfected HLA-A2^−^ LCL never elicited a response by CD8^+^ T cell clone D2-1-D2 (not shown). In addition, vvpp65-infected HLA-A2^−^ LCL alone in the absence of HLA-A2+ DC never elicited a response by CD8^+^ T cell clone D2-1-D2 (not shown). **4C**. Combined data from 3 separate cross presentation experiments using adult (*n = *18) and neonatal (*n = *14) HLA-A2^+^ DC. No significant differences were observed (p = 0.8587).

As a professional APC, DC can be distinguished from a non-professional APC in the ability to process and present exogenous cell-associated antigens on MHC class I molecules in a process known as cross presentation. Moreover, DC are crucial in priming naïve CD8^+^ T cells and this process most likely occurs with cross presented antigen *in vivo* [11]. Therefore, we sought to test the ability of DC to cross present cell-associated antigen. To assess the cross presentation pathway, we have established an experimental system that requires cell-associated antigen be processed and presented by an HLA-mismatched APC. In brief, HLA-A2-negative LCL are infected with vaccinia expressing the HCMV (vvpp65). Heat inactivation and UV irradiation are performed to prevent further viral replication and induce apoptosis in the LCL, respectively. HLA-A2-positive DC are then co-incubated with the virally infected and heat-treated LCL, and cross-presentation assessed via the release of IFN-γ by the cognate HLA-A2-restricted CD8^+^ T cell clone (D2 1-D2 specific for the HCMV pp65 peptide, NLVPMVATV). In each experiment we rule out the possibility of viral spread or direct presentation. For this control, HLA-A2-positive LCL, which unlike DC cannot cross present cell-associated antigen, are co-incubated with the vvpp65-infected HLA-A2-negative LCL either before or after virus inactivation. In all experiments, only the addition of vvpp65-infected HLA-A2-negative LCL that were not heat treated, to the HLA-A2-positive LCL, stimulated the CD8^+^ T cell clone D2-1-D2 (Figure 4a). To assess cross presentation, HLA-A2-positive DC from neonates and adults were incubated with the HLA-A2-negative LCL that were either infected with vaccinia (Figure 4b) or not and treated as described above. HLA-A2-negative DC did not cross present HCMV antigen pp65 (Figure 4b). HLA-A2-positive DC from neonates (*n = *14) and adults (*n = *18) were equivalent in their ability to cross present the HLA-A2-restricted antigen HCMV pp65 (Figure 4c, p = 0.8587).

## Discussion

Few reports exist to demonstrate whether or not antigen processing and presentation in neonates is functionally impaired. Studies using MLR to assess the MHC II pathway have yielded conflicting conclusions [12]–[14]. The most comprehensive study of MHC II antigen processing and presentation function in neonates was performed using cord blood-derived monocytes as APC. Here, a defect in the presentation, but not in the processing of MHC II antigens was demonstrated [15]. To the best of our knowledge, no report exists on the ability of human neonatal DC to process and present antigen via the MHC class I pathway.

CD8^+^ T cells are involved in the control of many viral, bacterial and protozoan pathogens [16]. Consequently, a defect in the CD8^+^ T cell-dependent recognition of intracellular pathogens could have a profound effect on the control of these pathogens. We functionally assessed presentation of MHC I antigens in neonatal DC and found no defect in the presentation of both MHC Ia and MHC Ib-restricted antigens. To evaluate the ability of neonatal DC to process and present cytosolic proteins using the well-defined classical MHC class I pathway, we delivered the antigen in the context of live virus infection. Here, antigens generated in the cytosol are degraded into peptides via the proteasome, transported via TAP into the ER, loaded onto newly synthesized MHC class I molecules, transported to the cell surface, where they can be recognized by CD8^+^ T cells. Our studies show that neonatal DC showed no defect in their ability process and present antigen via this classical MHC I pathway.

*In vivo*, DC that are not directly infected by pathogens can still prime CD8^+^ T cells through a mechanism known as cross priming [17]. Here, DC are uniquely capable of engulfing dying cells and presenting the exogenously acquired antigens onto MHC class I molecules using the ill-defined cross presentation pathway. More importantly, cross presented antigen is likely to be the primary mechanism by which CD8^+^ T cells are primed *in vivo* [11]. Consequently, defects in the cross presentation pathway could limit the acquisition of pathogen-specific adaptive immunity. Fonteneau et al. characterized the cross presentation pathway for cell-associated antigen in human DC from PBMC [18]. These authors showed that cross presentation of the cell-associated (either apoptotic or necrotic cells) influenza A matrix protein 1 required: phagocytosis or macropinocytosis, proteolysis by cathepsin D, the proteasome, TAP, transfer of the antigen from the phagosome to the MHC class I loading complex with newly synthesized class I molecules, and export of peptide-loaded MHC I to the cell surface. Only one step in this pathway, the ability of neonatal DC to take up necrotic or apoptotic LCL, has been assessed. In this case, Wong et al. found no defect in the ability of neonatal DC to take up exogenous cells [19]. Our studies support those conclusions and significantly advance our understanding of neonatal DC function. Ultimately, we found no defect in the ability of neonatal DC to perform the multiple sequential steps that culminate in the complex process of cross presentation.

Why are neonates defective in generating functional immune responses to pathogens *in vivo*? Our studies suggest that DC from neonates are intrinsically functional in all aspects of MHC I antigen processing and presentation. We have directly compared neonatal and adult DC using identical protocols that would allow us to discern even modest differences in APC function. Due to the limited numbers of DC that can be directly isolated from the blood or tissues of neonates, we were unable to address the function of neonatal DC directly *ex vivo*. Nonetheless, in keeping with our results, studies in mice that have evaluated purified dendritic cells have demonstrated few intrinsic differences between adult and neonatal immune cells [20], [21]. One difference however is that DC from neonates express decreased levels of IL-12p35 after LPS stimulation [12], [22]. Nevertheless, the biological effect of this deficiency is unclear. In *in vitro* studies, IL-12p70 production could be restored to adult levels with the addition of IFN-γ [23]. Furthermore, *in vivo*, neonates immunized in the presence of strong adjuvants can generate adult-like Th1 responses [4], [24], [25]. Thus, the impaired capacity of neonates to mount optimal immune responses may not lie with any specific cell type but may represent a difference in the environment of the neonate [3]. For example, Sun et al. showed that Th1 priming in neonatal mice could be altered by B cells that produced more IL-10 than B cells from adults [26]. Another example that illustrates an environmental differences in neonates is the finding that adenosine in neonatal blood can alter the production of TNF-α and IL-6 from neonatal monocytes in response to TLR stimulation [27]. Finally, in adults, but not neonates, cross-reactive T cells could provide some protection to distinct pathogens [28]. Thus, while the control of pathogens in neonates is deficient, we conclude that a defect in antigen processing and presentation function in neonatal dendritic cells is unlikely to represent the cause for diminished T cell responses in neonates.

## Materials and Methods

### Human subjects

Human subjects protocols and consent forms were approved by the Oregon Health & Science University Institutional Review Board. PBMC were obtained from normal adult donors by apheresis under written informed consent. Umbilical cord blood was obtained from healthy full-term neonates. Due to the fact that cord blood is considered medical waste, and that no identifying information was collected form the neonates, we obtained cord blood under an IRB-exempt protocol. Cord blood was collected in BD Vacutainer CPT and CBMC isolated per manufacturer's instructions. Both fresh and cryopreserved cells were used with equivalent results (data not shown).

### Cells, cell lines, and T cell clones

Monocyte-derived DC were prepared according to the method by Romani et al [29]. Briefly, PBMC or CBMC were resuspended in 2% Human Serum (HS) medium and allowed to adhere to a tissue culture flask at 37° C for 1 hour. Nonadherent cells were removed using three PBS washes with gentle rocking. Adherent cells were incubated with 10% HS medium containing 10 ng/ml of IL-4 (Immunex) and 30 ng/ml of GM-CSF (Immunex). After 5 days, cells were harvested with cell-dissociation medium (Sigma-Aldrich) and used as APC in assays. EBV-transformed B cell lines (lymphoblastoid cell lines, LCL) were generated in our laboratory using supernatants from the cell line 9B5–8 (American Type Culture Collection).

The CD8^+^ T cell clone D2-1-D2 recognizes the HCMV antigen pp65_(495–503) _NLVPMVATV in the context of HLA-A2 and was generated by limiting dilution analysis as previously described [30] except that T cells were stimulated using DC infected with the recombinant vaccinia virus expressing HCMV antigen pp65 (data not published). CD8^+^ T cell clone D160 1-23 recognizes an antigen derived from the cell wall of Mtb in the context of HLA-E and has been previously described [10]. CD8^+^ T cell clones were expanded using a rapid expansion protocol with anti-CD3 mAb stimulation [31].

### HLA-A*0201 typing

The assessment of HLA-A2 expression was performed by flow cytometry on PBMC or CBMC using the FITC-conjugated anti-HLA-A2 antibody (clone BB7.2) in comparison to a FITC-conjugated msIgG2 isotype control (clone G155-178) (BD Pharmingen).

### Viruses and Antigens

Recombinant vaccinia virus coding for HCMV pp65 under control of the vaccinia p7.5 promoter was used for DC infection. HCMV pp65 peptide nonamer _(495-503)_ NLVPMVATV was used (Genemed Synthesis, Inc.) for peptide loading of DC. The HLA-E restricted pronase cell wall antigen was derived as previously described [10].

### Infection of cells with recombinant virus

DC or LCL were pelleted, resuspended in medium (50 µl) and incubated with vvpp65 at a multiplicity of infection (moi) of 1.5 for 1 hour. Cells were then washed three times, resuspended in 10% HS RPMI and incubated overnight.

### Assays

ELISPOT. The detection of IFN-γ production by CD8^+^ T cells was performed by IFN-γ ELISPOT as previously described [9] with the exception that plates were read using the AID High Resolution Elispot Reader System with a Digital Firewire Camera, 1280x960 Color CCD Chip, QuadPack 2-axis precision stage, impact-protected, and Pentium PC computer with AID Elispot Software.

Cross presentation assay. The cross presentation assay described by Fonteneau et al. was used with several modifications [18]. As a source of cell-associated antigen we used HLA-A2-negative LCL that were either uninfected or infected with vvpp65 overnight. To prevent any direct vaccinia virus infection of the DC in the course of the experiment, we heated the LCL for 30 minutes at 56°C to inactivate the vaccinia virus following overnight infection [32]. We chose 30 minutes based on time course studies that showed that 30 minutes allowed for inactivation of the virus while maintaining preservation of the pp65 antigen (data not shown). To induce apoptosis, LCL were UV treated with 200 mJ^2^ and then incubated for 3 hours in medium at 37°C. LCL were then washed three times and added to HLA-A2^+^ DC (or HLA-A2^−^ DC as a control) in IFN-γ-coated ELISPOT plates at a ratio of 2:1 (LCL: DC) and incubated overnight. D2-1-D2 CD8^+^ T cell clones (10,000 cells/well), specific for NLVPMVATV in the context of HLA-A2, were added to ELISPOT wells with the DC and incubated overnight. Detection of IFN-γ by T cells was performed the following day.

#### Statistical Analyses

Prism software was used for all statistical analyses. To determine whether or not there were differences between neonatal and adult DC in experiments where peptide or DC number were titrated under limiting conditions, the average number of spots per well for each duplicate was plotted against the concentration of peptide or DC number respectively. Linear regression analysis was used to determine the slope of the line. It was then determined if the binomial probability for the number of spots was significantly different between adult and neonatal cells. Assessment of significant differences in cross presentation between adult and neonatal DC was performed using the Student's Unpaired T test.
